# Nasogastric/nasoenteric tube-related adverse events: an integrative review*

**DOI:** 10.1590/1518-8345.3355.3400

**Published:** 2021-01-08

**Authors:** Ana Paula Gobbo Motta, Mayara Carvalho Godinho Rigobello, Renata Cristina de Campos Pereira Silveira, Fernanda Raphael Escobar Gimenes

**Affiliations:** 1 Universidade de São Paulo, Escola de Enfermagem de Ribeirão Preto, PAHO/WHO Collaborating Centre for Nursing Research Development, Ribeirão Preto, SP, Brazil.

**Keywords:** Enteral Nutrition, Intubation, Gastrointestinal, Nursing, Patient Safety, Review, Patient Harm, Nutrição Enteral, Intubação Gastrointestinal, Enfermagem, Segurança do Paciente, Revisão, Dano ao Paciente, Nutrición Enteral, Intubación Gastrointestinal, Enfermería, Seguridad del Paciente, Revisión, Daño del Paciente

## Abstract

**Objective:**

to analyze in the scientific literature the evidence on nasogastric/nasoenteric tube related adverse events in adult patients.

**Method:**

integrative literature review through the search of publications in journals indexed in PubMed/MEDLINE, CINAHL, LILACS, EMBASE and Scopus, and hand searching, was undertaken up to April 2017.

**Results:**

the sample consisted of 69 primary studies, mainly in English and published in the USA and UK. They were divided in two main categories and subcategories: the first category refers to Mechanical Adverse Events (respiratory complications; esophageal or pharyngeal complications; tube obstruction; intestinal perforation; intracranial perforation and unplanned tube removal) and the second alludes to Others (pressure injury related to fixation and misconnections). Death was reported in 16 articles.

**Conclusion:**

nasogastric/nasoenteric tube related adverse events are relatively common and the majority involved respiratory harm that resulted in increased hospitalization and/or death. The results may contribute to healthcare professionals, especially nurses, to develop an evidence-based guideline for insertion and correct positioning of bedside enteral tubes in adult patients.

## Abstract (pt)

**Objetivo:**

analisar na literatura científica as evidências sobre eventos adversos relacionados à sonda nasogástrica/nasoentérica em pacientes adultos.

**Método:**

revisão integrativa da literatura realizada em abril de 2017 por meio da busca de publicações em periódicos indexados no PubMed/MEDLINE, CINAHL, LILACS, EMBASE e Scopus, e de buscas manuais.

**Resultados:**

a amostra foi composta por 69 estudos primários, principalmente em inglês e publicados nos EUA e Reino Unido. Eles foram divididos em duas categorias e subcategorias principais: A primeira categoria refere-se aos Eventos Adversos Mecânicos - complicações respiratórias; complicações esofágicas ou faríngeas; obstrução da sonda; perfuração intestinal; perfuração intracraniana e remoção acidental da sonda e a segunda, alude a Outros - lesão por pressão relacionada à fixação e conexão incorreta. A morte foi relatada em 16 artigos.

**Conclusão:**

os eventos adversos relacionados à sonda nasogástrica/nasoentérica são relativamente comuns e a maioria envolveu eventos adversos respiratórios que resultaram em hospitalização prolongada e/ou morte. Os resultados podem contribuir para que os profissionais de saúde, especialmente enfermeiros, desenvolvam diretrizes baseadas em evidências para a inserção e posicionamento correto de sondas enterais à beira do leito em pacientes adultos.

## Abstract (es)

**Objetivo:**

analizar en la literatura científica la evidencia con respecto a eventos adversos relacionados con sondas nasogástricas y/o nasoenterales en pacientes adultos.

**Método:**

una revisión integradora de la literatura realizada por medio de una búsqueda de artículos en publicaciones indexadas en PubMed/MEDLINE, CINAHL, LILACS, EMBASE y Scopus, al igual que por medio de búsquedas manuales, llevadas a cabo hasta el mes de abril de 2017.

**Resultados:**

la muestra estuvo compuesta por 69 artículos primarios, principalmente redactados en inglés y publicados en EE. UU. y en el Reino Unido. Se los dividió en dos categorías principales y subcategorías: la primera categoría se refiere a los eventos adversos mecánicos: complicaciones respiratorias; complicaciones esofágicas o faríngeas; obstrucción de la sonda; perforación intestinal; perforación intracraneal y extracción accidental de la sonda y el segundo, alude a Otro - lesión por presión relacionada con la fijación y conexión incorrecta. Se informaron fallecimientos en 16 artículos.

**Conclusión:**

los eventos adversos relacionados con sondas nasogástricas y/o nasoenterales son relativamente comunes y la mayoría implicó daños respiratorios que derivaron en internaciones prolongadas y/o en fallecimientos. Los resultados pueden contribuir al trabajo de los profesionales de la salud, especialmente los de Enfermería, en el desarrollo de una directriz basada en evidencias para la inserción y el correcto posicionamiento de los tubos enterales al lado de la cama en pacientes adultos.

## Introduction

The insertion of a nasogastric/nasoenteric tube (NGT/NET) is a common practice in acute and chronic care settings for the delivery of feeding and/or medications to patients of all ages^(^ 1 ^)^. This procedure may lead to adverse events (AEs)^(^ 2 ^)^ though, defined as an incident that resulted in patient harm or an injury that was caused by medical management or complications instead of the underlying disease and that resulted in prolonged hospitalization or disability at the time of discharge from medical care, or both^(^ 3 ^)^.

In the worldwide literature there are many reports of deaths associated with these tubes^(^ 4 ^)^. According to the Food and Drug Administration (FDA), from January 2012 to July 2017, there were 51 reports of pneumothorax related with NGT/NET placement. In most cases, urgent intervention was required, including needle decompression or chest tube insertion. Several of these events were associated with cardiopulmonary arrest and death^(^ 5 ^)^.

Another report from the National Patient Safety Agency (NPSA) showed that about 170,000 NGT/NETs are inserted *per* year for enteral nutrition and medication administration in the United Kingdom (UK). According to the NPSA, between 2005 and 2010, 21 deaths and 79 severe AEs caused by NGT/NET displacement were reported to the UK National Reporting and Learning System (NRLS), contributing to poor patient outcomes^(^ 6 ^-^ 7 ^)^.

In Brazil, severe and fatal NGT/NET-related AEs have been reported in the media. These events were mainly caused by misconnection, which resulted in the infusion of enteral nutrition into the vein^(^ 8 ^)^. Research conducted in the USA showed that up to 3.2% NGT/NET were inserted into the airway, resulting in pneumothorax and death^(^ 9 ^-^ 10 ^)^.

The inadvertent introduction of an NGT/NET into the tracheal tree results in patient discomfort, delayed eating, increased morbidity, mortality and the length of hospital stay. Nonetheless, other AEs related with NGT/NET insertion may occur, such as sinusitis, nasopharyngeal discomfort, nasal septum erosion, pressure injury, epistaxis and blood return through the tube in guidewire withdrawal. NGT/NET insertion can also cause pain, discomfort, vomiting and refusal of the procedure by the patients^(^ 9 ^)^. Thus, nurses should be aware of these risks in order to improve patient safety. In addition, the nursing care provided needs to be guided by Evidence-Based Practice (EBP)^(^ 10 ^)^.

Although NGT/NET-related AEs are common in hospitals settings with significant morbidity and mortality, the issue has not been extensively studied, especially in developing countries^(^ 11 ^)^. In Brazil, data on this topic is not available, however, it is observed that the use of these tubes are common in most Brazilian healthcare institutions^(^ 12 ^)^. Studies that aim to identify the types and the most frequent NGT/NET-related AEs can reduce that gap and the risks of harm caused to patients and lower overall cost of care^(^ 11 ^)^.

The lack of background studies on feeding tube-related AEs poses a difficult challenge, but at the same time highlights the importance of this study as a first essential step towards improving patient safety. Thus, the purpose of this integrative review was to analyze in the scientific literature the evidence on nasogastric/nasoenteric tube related adverse events in adult patients.

## Method

An integrative review was conducted in six phases^(^ 13 ^)^ in April 2017: selection of research question; literature search; categorization of data; analysis of studies included in review; interpretation and synthesis of outcomes and presentation of review. Beyond that, PRISMA guidelines^(^ 14 ^)^ were followed.

The search strategy for the articles and the research question were developed through the PICO strategy^(^ 14 ^)^: The population refers to adult patients (P); the intervention, to patients with SNG/SNE (I); there was no comparison group (C) and the outcome refers to the main adverse events (O). The following research question was asked: what are the main AEs in adult patients with SNG/SNE? To perform the search strategy, keywords were used that reflected the research question, with the boolean operators AND and OR.

The most relevant electronic databases for nurses were used: PubMed/MEDLINE, CINAHL, LILACS, EMBASE and Scopus. In addition, we used hand searching to check the reference lists of selected studies to see if these references included reports of other studies that could be eligible for this review.

The following search strategy was used: [(nasogastric tube) OR (feeding tube) OR (enteral tube) OR (enteral tube feeding) OR (nasogastric feeding tube) OR (nasoenteral tube)] AND [(medical errors) OR (adverse events) OR (adverse event) OR incidents OR incident OR mistakes OR mistake].

After completing the search, performed by two independent researchers, all articles were exported to EndNote Web Basic (Clarivate Analytics^®^) and duplicated articles were removed. The eligibility criteria were established based on the review question. Thus, primary studies were included which addressed NGT/NET-related AEs in adult patients (> 18 years); published in Portuguese, Spanish and English; and no publication time was applied. The excluded studies were: studies with adult patients with NGT/NET who did not address adverse events; studies evaluating adverse events related to the use of gastrostomy, jejunostomy and/or ileostomy; and types of publication such as literature reviews, conference abstracts, and book chapters.

Two independent reviewers extracted information from the selected articles using a standardized form^(^ 15 ^)^ based on the PICO formula^(^ 14 ^)^, also collecting data on: author(s), article date, country, type of research, sample definition, variable measuring and statistical analysis, main results and conclusions, as well as the level of evidence and recommendations by the authors. Thus, data from all of the selected studies were double extracted to check for consistency and any discrepancies which arose were discussed and resolved by the researchers, or were referred to the third reviewer for final decision.

The hierarchy of evidence classification proposed by Melnyk and Fineout-Overholt^(^ 14 ^)^ was used to evaluate the studies. This classification evaluates the level of evidence of each study and allows the researcher to analyze different types of methods.

Articles were analyzed and ranked according to the classification of AEs involving NGT/NET, as described by Blumenstein and colleagues^(^ 9 ^)^: *Mechanical Adverse Events* and *Others*.

The first major category, *Mechanical Adverse Events*, presented the following subcategories: respiratory complications; esophageal or pharyngeal complications; tube obstruction; intestinal perforation; intracranial perforation and; unplanned tube withdrawal. The second major category, called *Others*, included the following subcategories: pressure injury related to fixation and misconnection.

## Results

**Figure 1:**
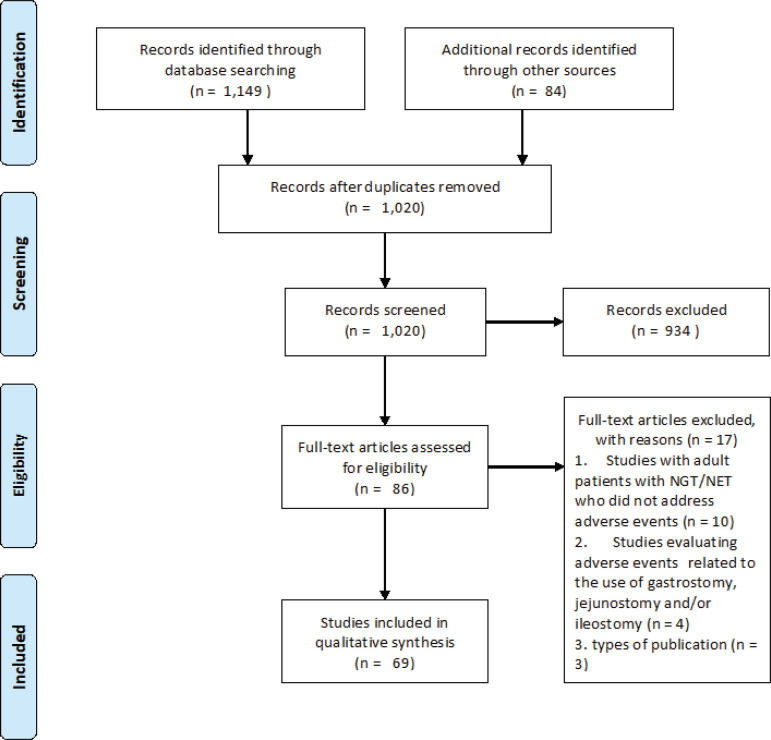
Identification and selection flow of articles included in the integrative review, through database search. Ribeirão Preto, SP, Brazil, 2018^(^ 16 ^)^

Sixty-nine articles were considered to meet the criteria for inclusion in this integrative review. Figure 1 depicts the stages of the screening process undertaken to reach this selection in PRISMA format.

Figure 2 gives an overview of the included articles according to general characteristics: author, year, country, language and level of evidence. The papers included predominantly came from the USA (n = 24) and UK (n = 8), were published in 2010 (n = 6) and 2012 (n = 6), in English language (n = 67) and were classified with level of evidence VI (n = 67). Among all articles included in this review, death was reported in 16 and the main cause was the incorrect handling of the tube. The results are presented according to categories and subcategories and key aspects of each paper are presented, with the main findings.

**Figure 2:** General characteristics of studies included in the review. Ribeirão Preto, SP, Brazil, 2018

Article n°	Author	Year	Country	Language	Level of Evidence
*Respiratory complications*					
1	Kearns, et al.^(^ 17 ^)^	2000	USA	English	II
2	Attanasio, et al.^(^ 18 ^)^	2009	Italy	English	VI
3	Neumann, Delegge^(^ 19 ^)^	2002	USA	English	II
4	Rassias, et al.^(^ 20 ^)^	1998	USA	English	VI
5	Lin, et al.^(^ 21 ^)^	2008	Taiwan	English	VI
6	McWey, et al.^(^ 22 ^)^	1988	USA	English	VI
7	Bankier, et al.^(^ 23 ^)^	1997	Austria	English	VI
8	Marderstein, et al.^(^ 24 ^)^	2004	USA	English	VI
9	de Aguilar-Nascimento, et al.^(^ 10 ^)^	2007	Brazil	English/Portuguese/Spanish	VI
10	Metheny, et al.^(^ 25 ^)^	2011	USA	English	VI
11	Marco, et al.^(^ 26 ^)^	2013	Spain	English/Spanish	VI
12	Sweatman, et al.^(^ 27 ^)^	1978	USA	English	VI
13	Vaughan^(^ 28 ^)^	1981	UK	English	VI
*Respiratory complications*					
14	Balogh, et al.^(^ 29 ^)^	1983	USA	English	VI
15	McDanal, et al.^(^ 30 ^)^	1983	USA	English	VI
16	Schorlemmer, Battaglini^(^ 31 ^)^	1984	USA	English	VI
17	Harris, Filandrinos^(^ 32 ^)^	1993	USA	English	VI
18	Thomas, et al.^(^ 33 ^)^	1996	USA	English	VI
19	Kolbitsch, et al.^(^ 34 ^)^	1997	Austria	English	VI
20	Metheny, et al.^(^ 35 ^)^	1998	USA	English	VI
21	Winterholler, Erbguth^(^ 36 ^)^	2002	Germany	English	VI
22	Kannan, et al.^(^ 37 ^)^	1999	UK	English	VI
23	Howell, Shriver^(^ 38 ^)^	2005	USA	English	VI
24	O'Neil, Krishnananthan^(^ 39 ^)^	2004	Australia	English	VI
25	Pillai, et al.^(^ 40 ^)^	2005	Canada	English	VI
26	Kawati, Rubertsson^(^ 41 ^)^	2005	Sweden	English	VI
27	De Giacomo, et al.^(^ 42 ^)^	2006	Italy	English	VI
28	Haas, et al.^(^ 43 ^)^	2006	Netherlands	English	VI
29	Freeberg, et al.^(^ 44 ^)^	2010	USA	English	VI
30	Lemyze, Brown^(^ 45 ^)^	2009	France	English	VI
31	Lo, et al.^(^ 46 ^)^	2008	USA	English	VI
32	Wang, et al.^(^ 47 ^)^	2008	Taiwan	English	VI
33	Ishigami, et al.^(^ 48 ^)^	2009	Japan	English	VI
34	Takwoingi^(^ 49 ^)^	2009	UK	English	VI
35	Chhavi, et al.^(^ 50 ^)^	2010	India	English	VI
36	Luo, et al.^(^ 51 ^)^	2011	China	English	VI
37	Shaikh, et al.^(^ 52 ^)^	2010	Qatar	English	VI
38	Sellers^(^ 53 ^)^	2012	UK	English	VI
39	Amirlak, et al.^(^ 54 ^)^	2012	USA	English	VI
40	Raut, et al.^(^ 55 ^)^	2015	India	English	VI
41	Andresen, et al.^(^ 56 ^)^	2016	Denmark	English	VI
42	Kao, et al.^(^ 57 ^)^	2012	China	English	VI
43	Leonard, et al.^(^ 58 ^)^	2012	Ireland	English	VI
44	Paul, et al.^(^ 59 ^)^	2013	USA	English	VI
*Esophageal or pharyngeal complications*					
45	James^(^ 60 ^)^	1978	UK	English	VI
46	Duthorn, et al.^(^ 61 ^)^	1998	Germany	English	VI
47	Isozaki, et al.^(^ 62 ^)^	2005	Japan	English	VI
48	Wu, et al.^(^ 63 ^)^	2006	Taiwan	English	VI
49	Campo, et al.^(^ 64 ^)^	2010	Spain	Spanish	VI
50	Sankar, et al.^(^ 65 ^)^	2012	UK	English	VI
51	Cereda, et al.^(^ 66 ^)^	2013	Italy	English	VI
52	Khasawneh, et al.^(^ 67 ^)^	2013	USA	English	VI
*Tube obstruction*					
53	Attanasio, et al.^(^ 18 ^)^	2009	Italy	English	VI
54	Cervo, et al.^(^ 68 ^)^	2014	Brazil	English/Portuguese	VI
55	Tawfic, et al.^(^ 69 ^)^	2012	Oman	English	VI
56	Van Dinter Jr, et al.^(^ 70 ^)^	2013	USA	English	VI
*Intracranial perforation*					
57	Wyler, et al.^(^ 71 ^)^	1977	USA	English	VI
58	Glasser, et al.^(^ 72 ^)^	1990	USA	English	VI
*Intracranial perforation*					
59	Freij, Mullett^(^ 73 ^)^	1996	UK	English	VI
60	Ferreras, et al.^(^ 74 ^)^	2000	Spain	English	VI
61	Genu, et al.^(^ 75 ^)^	2004	Brazil	English	VI
*Unplanned Tube Removal*					
62	Carrion, et al.^(^ 76 ^)^	2000	Spain	English	VI
63	Nascimento, et al.^(^ 77 ^)^	2008	Brazil	English/Portuguese and Spanish	VI
*Pressure Injury Related to Fixation*					
64	Güimil, et al.^(^ 78 ^)^	2010	Spain	Spanish	VI
*Misconnections*					
65	Ghahremani, Gould^(^ 79 ^)^	1986	USA	English	VI
66	Takeshita, et al.^(^ 80 ^)^	2002	Japan	English	VI
67	Roberts, Swart^(^ 81 ^)^	2007	UK	English	VI
68	Thorat, Wang^(^ 82 ^)^	2008	Singapore	English	VI
69	Millin, Brooks^(^ 83 ^)^	2010	USA	English	VI

### Category 1: Mechanical Adverse Events

#### Respiratory complications

Forty-four articles^(^ 17 ^-^ 59 ^)^ were included in this subcategory. This subcategory contained the largest number of articles, showing that respiratory complications were the most common NGT/NET-related group of AEs. In ten studies, death was reported due to incorrect insertion of the tube into the lungs^(^ 20 ^,^ 29 ^,^ 31 ^,^ 35 ^,^ 44 ^,^ 48 ^,^ 51 ^-^ 52 ^,^ 56 ^,^ 58 ^)^.

Respiratory AEs occurred mainly because of undue displacement of the NGT/NET to the respiratory tract and to the inconclusive results of the methods used to confirm the positioning of the distal tip of the tubes.

The most common respiratory AE was pneumothorax, followed by pleural effusion and bronchoaspiration related to enteral nutrition. In most cases, a chest drain was required. The authors also reported drainage of a considerable volume of enteral feeding, ranging from 300 mL^(^ 58 ^)^ to 900 mL^(^ 43 ^)^.

There were reports of NGT/NET-associated pneumonia (n = 9), and in these cases the patient required antibiotic therapy. The results of this subcategory are summarized in Figure 3.

**Figure 3:** Key features of the subcategory respiratory complications described in included articles. Ribeirão Preto, SP, Brazil, 2018

Article Number	Study Aim	Study Type	Main Results
1	To investigate the rate of VAP* and adequacy of nutrient delivery with gastric vs small intestinal feeding.	Prospective, randomized, controlled trial	ICU^†^ patients were observed for a period of 15 months. All patients needed mechanical ventilation and enteral nutrition. After tube insertion, all patients underwent radiography to confirm tube placement. Aspiration or VAP* was confirmed between patients with NGT^‡^ and NET^§^, but the difference was not significant.
2	To describe the management of patients treated with enteral nutrition and to identify complications and mortality.	Prospective observational study	From 108 patients included in the study, 45 used NET^§^, 62 used gastrostomy and one patient had jejunostomy. The following complications were observed: aspiration (15%); accidental removal (62%) and tube obstruction (11%). The mortality rate was 23% at one year and the average survival was 674 days.
3	To compare the outcomes of ICU^†^ patients fed through an NGT^‡^ vs. a nasal-small-bowel tube including the time from tube placement to feeding, time to reach goal rate, and adverse events.	Prospective randomized study	Sixty patients were randomized to receive gastric or small-bowel tube feedings. Adverse outcomes included witnessed aspiration, vomiting, and clinical/radiographic evidence of aspiration. There was no difference in aspiration events within groups.
4	To determine the type and incidence of pulmonary complications associated with the placement of narrow-bore enteral feeding tubes.	Prospective observational study	740 tubes were inserted and 14 cases (2%) of tube misplacement to the trachea and bronchi were identified. In all patients, auscultation was positive for rustling sounds, but radiography identified the incorrect positioning of the tip. Five patients suffered severe complications (pneumothorax) and two died.
5	To investigate the prevalence rate and influencing factors of pneumonia associated with long-term feeding in special care units for patients with persistent vegetative states (PVS).	Prospective observational study	Two hundred sixty subjects were chosen from three hospital-based special care units for patients with PVS and 10 nursing facilities for persons in PVS in Taiwan. Data were collected through chart review and observations. The factors associated with pneumonia were: length of hospital stay and enteral nutrition.
6	To report the case of 14 patients who had inadvertent tube misplacement, resulting in complications that included pneumothorax, empyema, mediastinitis, pneumonia, and esophageal perforation.	Retrospective observational study	Fourteen patients with a misplaced tube were selected over a period of 18 months. Of the 13 patients who had pulmonary complications, one had received enteral nutrition before confirmation by X-ray. Complications included pneumothorax, that required pleural drainage, and esophageal perforation.
7	To illustrate the radiographic spectrum of the intrabronchial malposition of gastric tubes and subsequent complications, and to discuss the role of radiography in the detection of such malposition.	Retrospective observational study	Over a period of 11 months, 14 cases of tube misplacement were recorded in the tracheobronchial tree. Of the 14 insertions, eight were performed blindly at the bedside and six by laryngoscope. Nine tubes were inserted in the right tracheobronchial tree and five in the left. Four patients had pleural perforation, with consequent pneumothorax and need for chest tube insertion. Another four patients developed pneumonia.
8	To determine whether a specialized feeding tube placement team had a beneficial effect on procedure-related pneumothorax.	Retrospective observational study	Over a three-year period, researchers analyzed reports of NET^§^ displacement to the tracheobronchial tree. Of the 4,190 patients included, 683 had respiratory adverse events associated with the tube; of these, nine suffered pneumothorax.
9	To investigate the use of radiographs, fluoroscopy, feeding tubes, and complications associated with blind feeding-tube placement.	Retrospective observational study	1,822 NET^§^ were inserted in 729 patients. In 23 patients, the tube was in the pulmonary position and nine had pneumothorax. There was a significant incidence of respiratory complications. Out of every 100 patients, three presented inadvertent tube positioning.
10	To determine the extent to which aspiration pneumonia are associated with feeding site (controlling for the effects of severity of illness, degree of head-of-bed elevation, level of sedation, and use of gastric suction).	Retrospective observational study	NGT^‡^/NET^§^ were inserted and the positioning was confirmed by radiography. The prevalence of pneumonia was significantly lower when the tube was located in the intestine, especially in the jejunum. This relationship remained when other variables were analyzed, including: disease severity and sedation level.
11	To determine the relationship between enteral nutrition feeding devices in patients admitted to the Internal Medicine Departments and the development of pulmonary complications (bronchial aspiration and aspiration pneumonia).	Retrospective observational study	2,767,259 hospital discharges were observed; of these, 0.92% were from patients receiving enteral nutrition via an enteral tube. These patients were found to be 15 times more likely to have bronchoaspiration and the risk of mortality was twice as high compared to patients who did not receive an enteral nutrition.
12	To describe two cases of accidental invasion of the trachea by esophageal tubes.	Case report	Patient underwent abdominal surgery due to dehiscence. Blind NGT^‡^ is inserted for gastric decompression. Upon arriving at the ICU^†^, the patient was restless and with increased respiratory rate. Arterial blood gases revealed hypoxemia. Mechanical ventilation had to be adjusted, but chest pectoral expansions were not observed and a radiograph showed that the tube was in the trachea.
13	To report a case in which passage of a narrow bore nasogastric tube into and through the right main bronchus and accidental soiling of the lung parenchyma with Clinifeed.	Case report	A 56-year-old man with head and neck cancer underwent surgery to remove the tumor. After surgery, NGT^‡^ was inserted and positioning was confirmed by radiography. About 400 mL of enteral nutrition was started. After the infusion, the patient presented dyspnea, cyanosis and increased heart rate. A new radiograph was performed and the tube was found to be located in the right main bronchus. The tube was removed and the patient required oxygen therapy.
14	To report three cases of pneumothorax attributable to misplacement of a commercially available mercury-weighted polyurethane feeding tube stiffened by a steel wire stylet.	Case report	A 73-year-old patient, after bypass surgery, had NET^§^ inserted for enteral nutrition. Positioning was confirmed by radiography, which revealed the presence of the distal tip in the right main bronchus. The tube was removed, however the patient had dyspnea and auscultation of the right lung revealed diminished sounds. A new radiograph was performed and pneumothorax was confirmed. The patient required thoracotomy to treat the adverse event and presented hemorrhage, coma, need for mechanical ventilation and, after seven weeks, he died.
15	To describe a case of massive intrapulmonary hemorrhage following the insertion of an NGT^‡^ into the tracheobronchial tree in an awake, alert, and cooperative patient.	Case report	An 82-year-old man suffered a car accident and was hospitalized after clavicle resection surgery. He was intubated with unstable vital signs and pulmonary edema. NGT^‡^ was required for gastric decompression. Initially, the tube was inserted uneventfully, but after a few minutes, blood was observed through the tube and vital signs decreased. Large amounts of blood returned through the tube. Radiography was performed and it was verified that the tube had crossed the left pleura. By laryngoscopy, the NGT^‡^ was removed and a left chest tube was required. About 1,500 ml of blood was drained. The patient continued with mechanical ventilation and required gastrostomy. Ninety days later, the patient was discharged home.
16	To report three cases of a potentially life-threatening complication associated with NGT^‡^/NET^§^.	Case report	In two cases, the patients were tracheostomized and required a feeding tube, but the tubes were accidentally removed. During insertion of the new tube, patients had respiratory distress and hypoxemia. The tubes were located in the pleura and pneumothorax was diagnosed. One patient died. The third case involved a patient already using a feeding tube, but it was removed accidentally, requiring a new insertion. The patient had a productive cough and the tube was removed. X-ray showed infiltration in the right lung median lobe and another tube was inserted.
17	To report a case of accidental activated charcoal instillation into the lung of a 30-year-old man being managed for a cyclic antidepressant overdose.	Case report	NGT^‡^ was inserted for activated carbon gastric lavage. Then, arterial puncture was performed and blood gases were within the normal range. The tube needed to be replaced without incident. 15 mL of activated charcoal were administered. The patient experienced a sudden change in oxygen saturation and vital signs though. Radiography revealed that the tube was in the right main bronchus and the patient was transferred to the ICU^†^ with the vital signs altered. He needed to be intubated and progressed to pneumonia.
18	To report an instance of the intrapleural administration of charcoal due to penetration of the pleura by a transbronchial nasogastric tube.	Case report	A 37-year-old woman was hospitalized for poisoning. During transport to hospital, NGT^‡^ was inserted for administration of 180 mL of activated charcoal for gastric lavage. The patient arrived at the hospital awake but lethargic. An initial x-ray revealed pneumothorax and insertion of the right bronchial tube. The tube was removed and a thoracotomy was performed, from which approximately 500 ml of liquid containing the coal was drained.
19	To report a case of pneumothorax caused by the improper placement of an NGT^‡^ in a tracheostomized patient after bilateral lung transplantation.	Case report	A 50-year-old man was admitted for lung transplant surgery. Due to postoperative complications, mechanical ventilation and tracheostomy were required. A NET^§^ was also inserted. There were three attempts and positioning was confirmed by auscultatory method. There was aspiration of yellowish residue. No radiography was performed because a chest tomography was scheduled. According to the exam, the tube was positioned in the lung and rupture of the right lower lobe pleura was identified. The tube was removed and a thoracotomy was performed.
20	To describe potentially disastrous outcomes associated with failure to determine when nasally inserted feeding tubes are improperly positioned.	Case report	Two cases were presented. In the first, uneventful NGT^‡^ was inserted in a 70-year-old patient with stroke and dysphagia. Placement was confirmed by two nurses using the auscultatory method; enteral nutrition was administered. After a few hours, the nurse observed that the patient was dyspneic and cyanotic and was transferred to the ICU^†^. The tube was found in the lung and the patient died of respiratory complications. In the second case, after 13 days of hospitalization, the patient required a new tube due to accidental removal. The confirmation method was performed by placing the distal tip of the tube in water. No blisters were observed and the enteral nutrition was started. After 3 hours, the patient had respiratory distress; radiography revealed that the tube was in the left main bronchus of the lung. Thoracic drainage was started to remove the fluid.
21	To report an instance of inadvertent placement of a standard NGT^‡^ into the left pleural space in a patient with right parietotemporal intracerebral hemorrhage and severe hemineglect on the left side.	Case report	A 69-year-old patient admitted for stroke was drowsy but able to communicate. NGT^‡^ was inserted for medication and feeding. There were no complications during insertion, and tube positioning was confirmed by abdominal auscultation. 100 mL of enteral nutrition were administered. After a few minutes, the patient had severe dyspnea. Radiography confirmed the positioning of the tube in the left bronchus, pleural effusion and pneumothorax. The tube was removed and patient intubation was required, followed by bronchoscopy and thoracotomy. In addition, the patient had pneumonia.
22	To report a case where the patient developed both tension pneumothorax and pneumomediastinum when an NGT^‡^ was inserted.	Case report	A 77-year-old woman was admitted to the ICU^†^ due to diabetic acidosis and subsequent left lower limb amputation. She required mechanical ventilation and, after three days, she was extubated. Six hours later, an attempt was made to insert an NGT^‡^, but there was difficulty during the procedure and the patient required oxygen supplementation. A new attempt was made, but without success. It was decided to insert the tube with the aid of lubricated biopsy forceps to act as a guide. The positioning of the tube was confirmed by aspiration of residue, but without success. Then the auscultation test was performed, and the result was negative. After a few minutes, the patient presented a reduction in oxygen saturation to 60%, increased blood pressure and tachycardia. The tube was removed and ventilatory support was provided. Radiography revealed right pneumothorax and the patient needed to be intubated again. A chest drain was also required.
23	To report a case of hydropneumothorax caused by inadvertent placement of a Dobhoff tube.	Case report	A 78-year-old woman was hospitalized due to maxillary carcinoma. She needed a NET^§^ for enteral nutrition. After tube insertion, the patient presented changes in vital signs (increased heart rate, increased respiratory rate and increased blood pressure). Arterial blood gas confirmed hypoxemia in ambient air and radiography revealed hydropneumothorax. The tube was inserted into the right lung. Enteral nutrition was started without confirming the tube positioning. Thoracoscopy was required to resolve the hydropneumothorax.
24	To report six cases of intrapleural NGT^‡^ insertion.	Case report	Six cases of elderly in the ICU^†^ with central nervous system dysfunction were reported. Of these, four were intubated and all had an NGT^‡^ inserted. The positioning of the tube was confirmed by radiography. In five patients, the tube was inserted into the right main bronchus and in one patient, the tube was inserted into the left bronchus. In five patients, the tube was immediately repositioned and, in one case, the patient received the enteral nutrition through a misplaced tube. Four elderly had pneumothorax.
25	To analyze the insertion of an NGT^‡^, though a common clinical procedure, and explore means to improve its safety.	Case report	An 80-year-old patient with previous bypass surgery required mechanical ventilation and remained in the ICU^†^ for a period of time. Patient required an NGT^‡^ for enteral nutrition and a radiograph was performed to confirm its positioning. The NGT^‡^ was located in the right pleural space. The tube was removed immediately and after two hours, a new radiograph confirmed pneumothorax.
26	To report three cases of nasopulmonary misplacement of the feeding tube in an ICU^†^.	Case report	One week after surgery, an 85-years-old man required an NGT^‡^ for enteral nutrition. Tube insertion occurred without complications and the tube positioning was confirmed by auscultation method and through the observation of yellowish residue. No misplacement was suspected. Enteral nutrition was started and after the infusion of 1,000 mL, the patient had decreased oxygen saturation, dyspnea and chest pain. Radiography revealed that the tube was in the right main bronchus, but there was no pneumothorax. The liquid was drained and the tube was removed by laryngoscopy. The patient had respiratory distress and a radiograph confirmed the pneumothorax; a chest tube was required. In the second case, a 70-year-old man with hypertension and peripheral vascular disease was admitted for lower limb amputation. He required an NGT^‡^ for enteral nutrition and insertion occurred uneventfully. Radiography confirmed the placement of the tube in the bronchus, with the extremity located in the pleura. Mild pneumothorax was diagnosed and the tube was removed by laryngoscopy. In the third case, a 65-year-old patient was admitted for pneumonia and was on mechanical ventilation. NGT^‡^ was inserted uneventfully. Positioning was confirmed by auscultation, which was positive. However, there was aspiration of one and a half liters of enteral nutrition though and fluid was found in the pleura. Radiography confirmed the positioning of the tube in the lung. The tube was removed, but the patient had sepsis.
27	To describe the bronchoscopic control of a significant and prolonged air-leakage, because of malposition of narrow-bore feeding tube, by placing a newly designed airway prosthesis with one-way valve into the corresponding segmental bronchus responsible for air-leakage source.	Case report	A 38-year-old woman diagnosed with bilateral pneumonia and respiratory failure was mechanically ventilated. An NGT^‡^ was inserted with the aid of an electromagnetic device. After a few hours, low saturation, tachycardia and hypotension occurred. Radiography revealed pneumothorax and a chest tube was inserted. The tomography showed that the tube was inserted into the tracheobronchial tree and that there was air leakage due to mechanical ventilation. The problem was solved by means of a valve, which was removed with subsequent extubation of the patient.
28	To report a serious complication of blind NGT^‡^ insertion in a 65-years-old female patient, which was overlooked and caused severe respiratory failure.	Case report	An NGT^‡^ was inserted and its positioning was confirmed by abdominal auscultation and radiography. On the following day, the patient presented cough, tachypnea and fever, with pleural effusion and collapse of the right lung lobe. Laryngoscopy confirmed the endotracheal positioning of the tube. This was removed without resistance. A radiograph revealed right pneumothorax and a thoracotomy with 900 mL drainage of the enteral nutrition was required.
29	To report 3 cases of severe pleuropulmonary complications after routine bedside placement of a narrow-bore enteral feeding tube.	Case report	Cases of severe pulmonary complications were reported after NET^§^ insertion. In two cases, the radiograph revealed a tube positioned in the lung, causing pneumothorax that needed to be drained. The third case dealt with a patient on mechanical ventilation whose tube was inserted into the lung with consequent pneumothorax. The patient died due to cerebral ischemia.
30	To report a case of an NGT^‡^ inadvertently positioned in the respiratory tract.	Case report	A 76-year-old man was admitted with a diagnosis of stroke. He needed a feeding tube due to risk of aspiration. The procedure was performed uneventfully and the patient had no complaints. The physician confirmed the positioning by the auscultation method and the enteral nutrition was released. After a few hours, the patient was transferred to the ICU^†^ due to acute respiratory failure. Radiographic examination revealed the placement of the tube in the lower lobe of the right lung.
31	To report a case of a misplaced NGT^‡^ into the pulmonary pleura.	Case report	A 50-year-old man was admitted to the emergency department. On the fourth day of hospitalization, an NGT^‡^ was inserted and confirmed by radiography. 750 mL of enteral nutrition were administered. The following day, the patient had shortness of breath and pleural effusion and pneumothorax were confirmed. The patient underwent thoracotomy and antibiotic therapy and he was discharged after 33 days.
32	To report a case of inadvertent NGT^‡^insertion into the right lower lobe bronchus.	Case report	A 79-year-old man with Chronic Obstructive Pulmonary Disease was admitted to the ICU^†^ and underwent mechanical ventilation. Subsequently, tracheostomy was performed. The patient was using an NET^§^ for enteral nutrition. A new tube was required and it was blindly inserted by the nurse in the ward. The position of the tube was confirmed by auscultation. Then, enteral nutrition was started. During the night, the nurse verified that the tube was wrapped around the patient's mouth and the tube was inserted again. Immediately after the enteral nutrition was administered, the patient coughed, and after several unsuccessful attempts, the nurse opened the tube and drained it. The following morning, a small amount of liquid was observed through the tracheostomy tube. An x-ray revealed that the tube passed through the tracheostomy balloon and into the right bronchus. The patient was tachypneic and did not respond to external stimuli and he was transferred back to the ICU^†^.
33	To report a case of NGT^‡^ inserted into the pleural cavity passing the trachea and left bronchi.	Case report	An 87-year-old woman hospitalized for pneumonia started an enteral nutrition due to lack of appetite. Two days after insertion of the tube, the patient presented with a decrease in general condition and dyspnea. Radiographs showed that the tube was located in the pleural cavity. Enteral nutrition was found in the left bronchus. The tube was removed and a pleural drain was introduced. The patient had pneumonia and pleuritis and after 12 days she died.
34	To report an unusual case of malpositioning of a fine bore NGT^‡^ into both main bronchi in a patient that was awake.	Case report	A 71-year-old woman with hypopharyngeal carcinoma required an NGT^‡^ after chemotherapy treatment. The tube was obstructed and a new one was required. There was no resistance during insertion, however the patient presented cough. The tube positioning was confirmed by auscultation. Next, a radiograph was performed and revealed that the tube was coiled in both bronchi. The tube was inserted into the left bronchus, bent and migrated to the right bronchus, and was therefore found in both major bronchi. Gastrostomy was required due to esophageal stenosis.
35	To report a case of accidental tracheal intubation of feeding tube in an intubated patient who developed respiratory distress a few minutes after test feed administration.	Case report	A 32-year-old man suffered a traffic accident with chest trauma, diaphragmatic rupture and fracture of left leg bones. He was operated and referred to the ICU^†^ and an NGT^‡^ was inserted for feeding. The insertion of the tube occurred uneventfully and its position was confirmed by auscultation. 100 ml of water was administered. After a few minutes, the patient presented respiratory disorder and decreased oxygen saturation, requiring mechanical ventilation. The positioning of the tube was verified again by laryngoscopy, which confirmed the positioning of the distal tip in the trachea.
36	To report six cases of tracheobronchial malposition of fine bore feeding tube in patients with mechanical ventilation.	Case report	Patient had cough and tachycardia during NGT^‡^ insertion and bronchoscopy confirmed inadequate positioning of the tube. In four patients, NGT^‡^/NET^§^ insertion was performed without complications and the test used to confirm the positioning was auscultation. Subsequently, bronchoscopy and radiography were performed to confirm possible pneumonia. Tests confirmed inadvertent placement of the tube. The sixth patient did not present cough during the insertion of the tube and the epigastric auscultation test was performed to confirm the positioning. A chest computerized tomography confirmed the placement of the tube in the tracheobronchial region. The patient died after 12 days due to the blood infection.
37	To report three cases of enteral feeding tube malpositioned into the respiratory system.	Case report	In the first case, a mechanically ventilated postoperative patient required an NGT^‡^ for gastric decompression. The spontaneous drainage bottle was filled with respiratory tidal volume. Radiography indicated that the NGT^‡^ was positioned in the left main bronchus. New NGT^‡^ was inserted by laryngoscopy. The second patient had diabetic foot, multiple organ dysfunction and sepsis and was admitted to the ICU^†^ after limb amputation. She was on mechanical ventilation and required a tracheostomy tube. She remained with enteral feeding via NGT^‡^. After five weeks with the tube, it needed to be replaced as it migrated to the left main bronchus. A new tube was inserted and the positioning was confirmed by radiography. The patient progressed to septic shock and died after 76 days of hospitalization. The third patient had spontaneous intraventricular bleeding and was admitted to the ICU^†^ with respiratory failure. NGT^‡^ was inserted and positioning was confirmed by radiography, which indicated the location of the distal tip in the right bronchus. The tube was immediately removed and another tube was inserted. The positioning of the new tube was confirmed by radiography.
38	To report a case describing false-positive NGT^‡^ placement confirmation tests in a patient with head and neck cancer, who was administered feed into lung parenchyma with significant morbidity.	Case report	A 54-year-old man with head and neck cancer was admitted to the ward for nutritional support. Blind NGT^‡^ was inserted and positioning was confirmed by pH test. Next, the administration of enteral nutrition began. The next day, the patient complained of nausea and 77% oxygen saturation in room air was observed. Radiography was performed and the positioning of the tube in the lung was confirmed. 540 mL of enteral nutrition were drained from the lung and antibiotic therapy was started.
39	To report two cases of pneumothorax following small-bore feeding tube insertion into the pleural cavity, resulting in pneumothorax.	Case report	In the first case, NET^§^ was inserted and the patient showed no signs of respiratory distress during insertion. However, the x-ray confirmed the position of the distal tip in the right main bronchus and consequent pneumothorax. In the second case, an NET^§^ was inserted in a patient on mechanical ventilation. During insertion, there was no change in oxygen saturation and the cuff remained inflated. However, the x-ray confirmed the placement of the tube in the left lung. Patient presented a decrease in saturation and blood pressure, a hypertensive pneumothorax and a chest tube were inserted.
40	To report a case of malposition of an NGT^‡^.	Case report	A 70-year-old man with Chronic Obstructive Pulmonary Disease was admitted for bypass surgery. After surgery, there was a need to insert an NGT^‡^, which occurred uneventfully. Positioning of the distal tip was confirmed by auscultatory method, but in the ICU^†^, radiography was performed before beginning the administration of enteral nutrition and medications. X-ray confirmed the placement of the tube in the right main bronchus.
41	To report the first documented fatality from pressure pneumothorax following NGT withdrawal.	Case report	An 84-year-old woman with dysphagia and risk of aspiration required a feeding tube. After insertion, the patient had difficulty breathing and the x-ray revealed positioning of the tube in the lung. The tube was removed, but the patient died after one hour. Necropsy showed cause of death: pneumothorax after tube withdrawal.
42	To report a case of severe acute respiratory distress syndrome induced by bronchopleural fistula due to malposition of NGT^‡^.	Case report	A 67-year-old man received enteral nutrition and, after 17 hours, severe cough and decreased oxygen saturation was observed. The patient was transferred to the ICU^†^ and required mechanical ventilation. The patient had a cough with thick yellow fluid and bronchoscopy. The examination showed the presence of enteral nutrition in the bronchi, and pulmonary lavage was performed. Radiography confirmed pleural effusion, requiring several pulmonary lavages, but not enough improvement in oxygen saturation. After several daily washes, saturation was normalized and thoracentesis was performed to remove pleural fluid.
43	To report two cases of NGT^‡^ placement which resulted in significant morbidity from a common procedure.	Case report	An 88-year-old woman, admitted by stroke, required an enteral feeding tube. Two days after the insertion, it needed to be replaced with another one. Positioning was confirmed by the epigastric auscultation method. Soon after, the patient showed agitation and radiography confirmed the positioning of the tube in the right main bronchus and pneumothorax. A chest tube was introduced, but the patient progressed to pneumonia. Subsequently, the medical team chose to feed her via gastrostomy. A 73-year-old patient was admitted to the geriatric ward because of circulatory complications. NGT^‡^ was inserted for feeding and the position was confirmed by radiography. Enteral nutrition was then released. After five hours, the patient presented respiratory impairment. A new x-ray confirmed that the tube was positioned in the lung and that there was about 300 mL of liquid, as well as abscess and pleural effusion. The fluid was drained and the patient was treated with antibiotics. This adverse event resulted in increased length of hospital stay and death after six months.
44	To report a case of a right-sided malpositioned NGT^‡^ which caused a pneumothorax only on its removal.	Case report	An 85-year-old female with advanced dementia was admitted due to severe dehydration caused by poor appetite. Intravenous solutions were infused and NGT^‡^ was inserted for enteral feeding. Initially, the procedure was uneventful, but a cough was observed. A radiograph was taken and it showed that the tube was positioned in the right main bronchus. The NGT^‡^ was removed. Then, the patient evolved to thoracic discomfort and the second radiograph found pneumothorax. The patient required supplemental oxygen for two days.

* VAP = Ventilator-associated pneumonia;

† ICU = Intensive Care Unit;

‡ NGT = Nasogastric tube;

§ NET = Nasoenteric tube

#### Esophageal or pharyngeal complications

In this subcategory, eight case reports^(^ 64 ^-^ 67 ^)^ were included; the authors described AEs related to the esophageal and/or pharyngeal insertion of the NGT/NET. In two case reports, the event led to vocal cord paralysis and laryngeal harm^(^ 62 ^,^ 64 ^)^.

In one article, the authors described the case of a patient with perforation of the nasopharynx, anterior carotid artery and internal jugular vein after insertion of the tube because its distal tip crossed the parotid gland^(^ 67 ^)^. Death was reported in two articles due to nasogastric tube syndrome^(^ 62 ^)^ and fatal massive hemorrhage caused by nasogastric tube misplacement^(^ 63 ^)^. These results are summarized in Figure 4.

**Figure 4:** Key features of the NGT/NET-related adverse events described in included articles. Ribeirão Preto, SP, Brazil, 2018

Article Number	Study Aim	Study Type	Main Results
*Esophageal or pharyngeal complications*			
45	To report a case that illustrates circumstances in which a narrow bore NGT* was misplaced and where there could have been serious consequences.	Case report	An NGT* was inserted in a 66-year-old patient. There was resistance during the insertion and a new attempt was made. During the pH check, results were found outside the normal range. A radiograph was performed and esophageal perforation was detected. The tube pierced the mediastinum and punctured the pleura. The tube was removed and the patient received antibiotic treatment.
46	To show that an acute and potentially life-threatening situation may arise after uneventful passage of an NET^†^.	Case report	After blind insertion of an NET^†^, a 56-yearl-old woman had large nasal bleeding. The tube punctured the right internal jugular vein and traversed the superior vena cava and the right atrium. She was quickly intubated to ensure patent airway and two liters of blood were drained. Vasoactive medication and intravenous blood infusion were also started. Patient was transferred to another hospital.
47	To describe the clinical histories of two representative cases among the four patients and discuss the etiology of this variant form of NGT* syndrome.	Case report	After prolonged use of an NGT*, one patient developed laryngeal stridor and severe vocal cord paralysis, as evidenced by laryngoscopy. The patient progressed to severe respiratory disease and died. In the second case, the patient presented laryngeal stridor, vocal cord paralysis and glottic space crack after NGT* removal. After two months, the patient presented exacerbated stridor and died due to respiratory failure.
48	To report a case of fatal hemorrhagic shock immediately after NGT* insertion in a patient undergoing debridement by video- assisted thoracoscopic surgery for mediastinitis.	Case report	An NGT* was inserted in a 70-year-old woman. During transport to the ICU^‡^, the tube was accidentally pulled out and it was replaced by the surgeon. After three attempts, there was a large amount of bleeding through the tube and vital signs changed dramatically, with a decrease in blood pressure and heart rate. Resuscitation maneuvers were initiated and the opening of the ribcage was necessary for direct cardiac compression. Four liters of blood were drained. Endoscopy revealed esophageal perforation, which caused bleeding. The tube was removed by endoscopy. Two days after the event, the pupils became fixed and the patient died.
49	To report a case of a 70 year-old woman who presented acute dyspnea, requiring emergency tracheotomy following prolonged nasogastric intubation.	Case report	Patient required an NGT* for enteral nutrition. After five weeks, a new tube was needed and after the insertion, the patient presented laryngeal stridor, vocal cord paralysis and arytenoid edema. Urgent tracheostomy was required. The tube was removed and parenteral nutrition was started. The patient gradually recovered vocal cord mobility and was diagnosed with Nasogastric Tube Syndrome.
*Esophageal or pharyngeal complications*			
50	To present a case that highlights the benefits of carrying out an X-ray to confirm the position of a nasogastric tube.	Case report	An NGT* for enteral nutrition was inserted in a 50-year-old man. After two weeks, the tube was inserted several times due to accidental removal. On one occasion, the patient reported traction of the tube while sleeping, but the tube was not found. A new tube was inserted and its tip position was confirmed by radiography. The x-ray revealed that the tube had been inserted into the left main bronchus. It also revealed that the first tube was in the hypopharynx region and the other end was in the stomach. The "lost" tube was removed by esophagoscopy and there were no complications to the patient.
51	To report an unexpected cause of malfunctioning NGT* due to non apparent misplacement.	Case report	An NGT* was blindly inserted in a 68-year-old man and positioning was confirmed by abdominal radiography. Enteral nutrition was started and the patient had vomiting. The attending physician reviewed the x-ray showing positioning of the tube in the esophagus.
52	To present a case of nasopharyngeal perforation caused by electromagnetically visualized feeding tube system.	Case report	NET^†^ was inserted with an electromagnetic device in a 50-year-old woman and mechanically ventilated. Resistance occurred during insertion; patient showed signs of respiratory distress and right side dilation of the face. A tomography showed perforation of the right nasopharynx. The tube traversed the anterior carotid artery, the internal jugular vein, and the parotid gland.
*Tube obstruction*			
53	To describe the management of patients treated with enteral nutrition and to identify complications and mortality.	Prospective observational study	From 108 patients included in the study, 45 used NET^†^, 62 used gastrostomy and one patient had jejunostomy. The following complications were observed: aspiration (15%); accidental removal (62%) and tube obstruction (11%). The mortality rate was 23% in one year and the average survival was 674 days.
54	To identify adverse events related to enteral nutrition in hospitalized patients	Longitudinal exploratory study	46 patients were observed and the most common adverse events were: accidental removal (43%) and tube obstruction (21%). Nausea and vomiting were also recorded.
55	To report a case of a patient who developed an esophageal bezoar due to malpositioning of an NGT*.	Case report	An NGT* was inserted into a 20-year-old patient. Positioning was confirmed by auscultation. Patient had aspiration pneumonia and the nurse found tube obstruction. A new tube was inserted and, again, obstruction was detected due to bezoar.
*Intestinal perforation*			
56	To describe a case where insertion of an NGT* caused intestinal perforation in a patient who had previously undergone Roux-en-Y gastric bypass.	Case report	An orogastric tube was inserted in a 59-year-old patient for gastric decompression. Positioning was confirmed by radiography. In the ICU^‡^, the tube was replaced by an NET^†^. On the 28th day, a new NET^†^ was inserted and 11 days later, a distended abdomen and absence of airborne noises were observed. The patient progressed to clinical worsening and on the 39th day, the patient died. At necropsy, intestinal perforation was found in the bypass region caused by the insertion of the last tube.
*Intracranial perforation*			
57	To describe a case in which a patient who had suffered severe facial fractures erroneously had an NGT* placed in the intracranial cavity.	Case report	A 34-year-old woman falls from the height of a building and suffers head and neck trauma. An NGT* was blindly inserted for gastric decompression and minutes later the patient had dilated pupils, ataxic breathing, and flaccid body. Radiography revealed that the tube surpassed the cribriform plate and that the distal tip was inserted into the intracranial cavity. The patient's condition deteriorated and she died after one hour.
58	To report a case of inadvertent intracranial complication directly related to the placement of an NGT* in a patient who had no history of head trauma.	Case report	An NGT* was inserted into a conscious and oriented 45-year-old woman with no previous history of head injury. During the insertion, there was return of live blood in the tube. The procedure was continued and the auscultation test was negative. The tube was removed and the patient became irresponsive. Computed tomography revealed subdural pneumocephalus of the skull and sinusitis in the frontal sinuses, with air collections.
59	To report a case of inadvertent intracranial placement of an NGT* in a non-trauma patient.	Case report	An NGT* was inserted into a 59-year-old woman. Three attempts were made and blood returned in all. In the third attempt, an x-ray was performed, which found the presence of the tube in the brain. The tube was removed, but the patient died of sepsis.
60	To describe a case of severe craniofacial fracture in which an NGT* was positioned intracranially.	Case report	An NGT* was inserted into a 38-year-old man with skull and facial bone base fractures. There were no clinical signs showing NGT* misplacement. After computed tomography, it was found that the tube was located in the cranial fossa.
61	To describe a case of severe craniofacial fracture in which the NGT* was positioned intracranially.	Case report	NGT* was inserted in a 53-year-old man with polytraumas. Skull base fracture was found and computed tomography revealed traumatic subarachnoid hemorrhage. The exam also revealed that the NGT* crossed the cribriform plate and reached the posterior cranial fossa. The tube was removed and the patient was transferred to the ICU^‡^. A drain was installed as well as a transducer for intracranial pressure monitoring. The next day, the patient presented hemiplegia on the right. The patient was only discharged after 80 days of hospitalization with neurological complications.
*Unplanned Tube Removal*			
62	To characterize the rates of accidental removal of endotracheal tubes, vascular catheters, and nasogastric tubes in the critically ill patient.	Prospective observational study	In total, 532 ICU^‡^ patients were included and 913 NGT* were inserted. Regarding accidental withdrawal, 312 cases were reported, and the most common reason was withdrawal by the patient her/himself.
63	To characterize adverse events in ICU^‡^, Semi-Intensive Care Units and Inpatient Units, regarding nature, type, day of the week and nursing professionals/patient ratio at the moment of occurrence; as well as to identify nursing interventions after the event.	Retrospective observational study	The main adverse events were related to NGT*/NET^†^: 69.6% were caused by accidental removal and 54.10% by tube obstruction.

* NGT = Nasogastric tube;

† NET = Nasoenteric tube;

‡ ICU = Intensive Care Unit

#### Tube obstruction

In this subcategory, three articles were included and the observational studies pointed out that the main AE was tube obstruction. In one study^(^ 18 ^)^, the rate of tube obstruction was 11%, whereas in another^(^ 68 ^)^ the rate was 21%. In the case report, medication administration was needed to unclog the tube and the cause of the obstruction was the concomitant administration of enteral feeding and medications. The drug-nutrient interaction resulted in bezoar formation that obstructed the tube lumen^(^ 69 ^)^ (Figure 4).

#### Intestinal perforation

This subcategory included one case report^(^ 70 ^)^. The authors described the case of a patient who died due to intestinal perforation after NET insertion. At necropsy, intestinal perforation was found in the bypass region caused by insertion of the last tube.

#### Intracranial perforation

In this subcategory, five case reports^(^ 71 ^-^ 75 ^)^ were included and the majority were accident victims with a resulting skull base fracture. Due to the rupture of the cribriform plaque, the NGT penetrated the intracranial region^(^ 71 ^,^ 74 ^-^ 75 ^)^. Death was reported in two articles^(^ 71 ^,^ 73 ^)^. The result was summarized in Figure 4.

#### Unplanned tube removal

In this subcategory, two articles were grouped^(^ 76 ^-^ 77 ^)^. In one, the authors calculated the rate of tubes removed accidentally^(^ 76 ^)^. In the other article, the authors performed a retrospective study and found that the most frequent AE was the unplanned tube removal^(^ 77 ^)^. The most common cause was removal by the patient^(^ 76 ^-^ 77 ^)^ (Figure 4).

### Category 2: Others

#### Pressure injury related to fixation

In this subcategory, a prospective observational study^(^ 78 ^)^ was included. The study showed that the incidence of NGT/NET-related pressure injury was 25.2%, related mainly to tube fixation (Figure 5).

**Figure 5:** Key features of the Category 2: Other Incidents described in included articles. Ribeirão Preto, São Paulo, Brazil, 2018

Article Number	Study Aim	Study Type	Main Results
*Pressure Injury Related to Fixation*			
64	To find out the incidence of patients with nasal pressure ulcer, study the risks factors for its development and find the predictable variables.	Prospective observational study	Pressure injury related to NGT*/NET^†^ fixation was found in 25.2% of all patients included in the study (n = 115).
*Misconnections*			
65	To determine if critically ill adult patients could be safely intubated at their bedside, and which complications might occur when the procedure is not controlled fluoroscopicaly.	Prospective observational study	314 patients were enrolled in the study who required an NET^†^. From those: - the tube was positioned in the airway in 7 patients (2.22%). - The tube was positioned in the esophagus in 8 (2.54%) patients and it resulted in bronchoaspiration. - the tube entered the stomach, but made a turn and returned to the esophagus in 2 patients (0.64%). - An AE occurred due to mercury leakage in the stomach of the distal end of the tube. This event occurred because the tube was wrapped around the stomach, which resulted in increased pressure and disconnection of mercury weight from the distal tip. The tube was removed and the mercury was gradually eliminated by the gastrointestinal system.
66	To report a case of a 77-year-old woman who had an inadvertent fatal administration of enteral feed via a venous catheter.	Case report	Patient received inadvertent infusion of enteral nutrition into the bloodstream via central venous catheter. Patient presented tachycardia, dyspnea and death after six hours of the event.
67	To report a case of a 74-year-old woman who had enteral formulas given by the wrong route.	Case report	Medication was administered into the patient's vein, who showed a rapid decline in consciousness and in respiratory function. The patient was intubated and required thoracic drainage. The patient evolved to sepsis and required tracheostomy. She was clinically stable and after a few days and she was extubated. She was discharged after eight weeks of the event.
68	To report a case of a 48-year-old man who had gastric acid burns because of a disconnected nasogastric tube.	Case report	Stroke patient restricted to bed presented 8% of burned body due to tube disconnection. The patient recovered after skin grafting.
69	To report a case of enteral feeding tube misconnection reported to the FDA^‡^.	Case report	High-flow oxygen was accidentally connected to the NGT*. The patient underwent emergency surgery to repair the gastric perforation and colonic serosal tear resulting from the improper connection.

* NGT = Nasogastric tube;

† NET = Nasoenteric tube;

‡ FDA = Food and Drug Administration

#### Misconnections

We included five articles^(^ 79 ^-^ 83 ^)^ in this subcategory that portrayed the AE caused by misconnection. In one article, the authors reported the case of a patient with NGT and who presented 8% of burned body surface due to the extravasation of gastric juice after accidental tube disconnection^(^ 82 ^)^. In two studies, the patients used a central venous catheter and an NGT/NET. The nurse inadvertently connected the enteral cable set to the central venous catheter. One patient received enteral feeding in the bloodstream and died^(^ 80 ^)^; in another study, the patient received oral medications in the bloodstream, requiring orotracheal intubation. The patient was discharged after eight weeks^(^ 81 ^)^ (Figure 5).

There was a report of a patient on mechanical ventilation who received a high oxygen flow in the stomach due to misconnection of the tube to the oxygen flow meter. The patient required surgery to repair the gastric perforation caused by the misconnection^(^ 83 ^)^.

## Discussion

Most NGT/NET-related AEs identified in this integrative review involved respiratory complications. However, other events were also identified, such as intestinal and intracranial perforation, tube obstruction, esophageal harm, unplanned tube removal, fixation-related pressure injury, and misconnection. In addition, 16 articles reported patient death as a consequence of the event.

Although considered a relatively simple and innocuous procedure, bedside insertion of an NGT/NET is associated with severe AEs. In addition, more than 88% of nurses are using non-evidence based methods for verification of NGT placement leading to serious patient harm^(^ 84 ^)^ and raising an important safety concern.

AEs related to misplaced NGT/NET can range from pneumothorax, requiring chest tube placement, to profound chemical pneumonitis and respiratory distress syndrome^(^ 84 ^)^. In this integrative review, the largest number of articles was grouped into the subcategory Respiratory complications, revealing that this was the main AE related to this medical device. Previous studies showed that patients had an NGT/NET inserted into the airway, resulting in pneumothorax. This event can be catastrophic, especially in critically ill patients, and according to the articles, pneumothorax occurred mainly because the tube was placed blindly at the patient bedside.

Respiratory AEs may also occur due to failure to recognize when an NGT/NET has changed position^(^ 84 ^)^ and when the methods used to verify its placement are inconclusive^(^ 85 ^)^. Healthcare professionals should be aware of these potential risks, especially in critically ill patients^(^ 86 ^)^.

Despite the risks, no universal standard of practice exists for bedside verification because each method has limitations^(^ 84 ^)^. There is a consensus among the international guidelines though about practices that should never be used to confirm blindly inserted feeding tubes, which include: auscultation^(^ 1 ^,^ 84 ^)^, visual inspection of fluid from the tube^(^ 1 ^,^ 84 ^)^, and observation of water bubbles^(^ 84 ^)^.

An x-ray, when properly performed and interpreted, is the most accurate method for distinguishing between gastric and pulmonary placement of a newly inserted NGT/NET and it is generally supported for high-risk patients (such as patients who are critically ill or have an altered level of consciousness or diminished or absent gag reflex)^(^ 1 ^)^. The existing British National Health Services (NHS) Improvement safety guideline recommends the pH method as first-line testing for initial NGT placement though. According to this guideline, pH ≤ 5.5 is considerate safe and this range excludes placement in the respiratory tract^(^ 87 ^)^. For blind inserted NET, an x-ray remains the safest method to confirm the tip position.

Nurses should also be aware that once correct NGT placement is confirmed, the exit site from the patient’s nose or mouth should be immediately marked and documented. In addition, after feedings are started, tube location should be checked at four-hour intervals^(^ 88 ^)^.

Several technologies are available to assist nurses during NGT/NET insertion, but specialists argue that lack of availability of special testing equipment, such as carbon dioxide detectors and enteral access devices, in routine clinical settings is a limiting factor for their use, and the evidence base for their accuracy has not been established^(^ 1 ^)^. Thus, based on research and best practices from the NHS Improvement and the New Opportunities for Verification of Enteral Tube Location (NOVEL) project from the American Society for Parenteral and Enteral Nutrition (ASPEN), evidence-based best practices to verify NGT placement include: nose-ear-mid umbilicus measurement (NEMU) every time an NGT is inserted (from the tip of the patient’s nose to the earlobe and from the earlobe to the point midway between the xiphoid process and umbilicus), pH testing, x-rays, and critical-thinking skills^(^ 84 ^)^.

Although patient outcomes are more severe when enteral nutrition is given to the lungs, esophageal/pharyngeal AEs can also cause significant harm, such as perforation of the pharynx, carotid artery and internal jugular vein. Among the AEs in the pharyngeal region, the Nasogastric Tube Syndrome was mentioned in two case reports included in this review. This syndrome consists of bilateral vocal cord paralysis accompanied by supraglottic edema. Despite being a rare syndrome, it can be considered fatal^(^ 89 ^)^. The triggering mechanism of the syndrome is the passage of the tube through the muscles present in the vocal cord region and the compression of bones against the spine, causing an inflammatory process. In addition, the primary symptoms are nonspecific, such as irritation and pain. However, in the Nasogastric Tube Syndrome, laryngeal stridor and vocal cord paralysis also occur. With proper treatment, the patient can slowly regain normal vocal cord mobilization^(^ 89 ^)^.

The pharyngeal complications identified in this review occurred because NGT/NET were blindly inserted at the bedside, making it impossible for the healthcare professional to visualize the route of the tube into the gastrointestinal tract (GIT). Due to resistance during insertion, caution is necessary in order to avoid perforation of internal organs. In addition, nurses should monitor patients with NGT/NET to manage risks and improve patient outcomes.

Another AE found in this study was intestinal perforation, which can be fatal due to subsequent infection. In one study, intestinal perforation occurred because the NET deviated at the bypass site. This does not mean that patients undergoing this type of surgical intervention are more prone to AEs. It should be emphasized, however, that the feeding tubes were designed for insertion in normal GIT. Therefore, in individuals with a different tract, both due to congenital defects and previous surgeries that caused anastomosis, the tubes should be inserted with the help of technologies that permit their real-time view^(^ 90 ^)^. The electromagnetic device is one such technology. A Brazilian study found that post-pyloric insertion of the tube guided by this device was faster and more efficient when compared to pH-testing^(^ 91 ^)^.

Another technology available to assist the practitioner during tube insertion is fluoroscopy. This method enables the tube path to be delineated through water-soluble contrast. Thus, this method allows the NGT/NET to be monitored in real time during insertion. The disadvantage is that it cannot be performed at the bedside, however, researchers reported that the success rate was 90% when using fluoroscopy guidance^(^ 92 ^)^.

The endoscopy method may also be appropriate for post-pyloric tube positioning. This method permits real-time visualization during tube insertion. Although considered a high-cost procedure that requires intravenous sedation, researchers showed a high success rate (98%)^(^ 93 ^-^ 94 ^)^.

Feeding tube insertion into the intracranial region is one of the most severe feeding tubes-related AEs. In the case reports included in this review, the tube penetrated the intracranial region due to the rupture of the cribriform plaque. Treatment in these cases consists of removing the tube and initiating antibiotics therapy when necessary. The mortality rate can be up to 60%, so extra precautions should be considered when inserting feeding tubes in patients with cranial fractures^(^ 75 ^)^. In these patients, the oropharyngeal route should be adopted, preferably with the help of an endoscope or laryngoscope, because they permit a direct view of the tube path. It is also recommended to use more calibrated tubes, preventing them from bending or being inadvertently diverted to an unwanted region^(^ 95 ^)^.

Regarding tube obstruction, in most cases, it is an event that occurs due to errors in tube handling, so nurses need to be attentive and follow the manufacturer’s norms^(^ 96 ^)^. In one study, the obstruction was related to the formation of bezoar. Bezoars are non-digestive conglomerates that accumulate in the tube, which may be, for example, medicines, enteral feeding and food residues. In the case presented in this integrative review, bezoar was formed by the enteral formulas that accumulated along the tube. Obstruction prevention requires safe practices in the handling of feeding tubes, which include checking the compatibility of the grinding and medication administration, crushing oral formulas until a fine powder, ensure feed is stopped prior to medication administration, administering medicines separately and washing the tube between administrations^(^ 97 ^-^ 98 ^)^.

In relation to unplanned feeding tube withdrawal, it can occur for several reasons, such as the health team itself, the patient and/or caregiver. Some interventions implemented in an ICU of a hospital in Rio de Janeiro, Brazil, decreased the frequency of unplanned removal, such as the evaluation of the presence of delirium or dementia, guidelines to the caregivers and mechanical restraint, when prescribed by the medical team, until agitation decreases^(^ 99 ^)^.

As a nursing intervention to reduce the risk of unplanned removal, researchers developed a technique that should be performed prior to the insertion of NGT/NETs. The technique consists of using two NETs, one inserted in each nostril. The two tubes are inserted into the oropharynx so that the distal tips progress to the oral cavity. The practitioner then ties the two distal tips with a knot, pulls one of the proximal tips until the knot protrudes through the nostril. About 16 cm of tube should be exposed, starting from the nostril. Thus, the NGT/NET is inserted through the GIT and tied underneath the prepared mechanism; one should leave about two inches for patient comfort and then fix the tube with hypoallergenic adhesive tape^(^ 100 ^)^. An English study found that, when this method is used, it saves approximately £3,288 *per* year. Therefore, this technology may be economically feasible because unplanned tube removal requires new insertion and new material consumption^(^ 101 ^)^.

The insertion of NGT/NET can be considered the most physiological means to permit enteral feeding in patients who are unable to receive oral feeding. These devices can cause discomfort and complications for the patient though, due to the material they are made of and to the nasal access. Nasal pressure injury is not a severe complication, however, this AE is avoidable through good nursing practices, such as the movement of the tube every 24 hours. The feeding tube is an external agent, therefore, its long dwelling time, without daily movement, can cause injury and discomfort^(^ 78 ^)^.

Continuing nursing education may help to reduce this type of AE. Nurses should be trained to move the tube in order to reduce pressure injury. In addition, preference should be given to the use of flexible and lubricated tubes^(^ 102 ^)^.

Misconnections can lead to serious AEs for patients and be related to medical devices (such as the NGT/NET itself, venous catheters and oxygen therapy devices). It was noticed that several devices had a Luer connection and that the enteral cable set could be connected to the venous access for example. To reduce these AE, the International Organization for Standardization (ISO) has implemented standards for connectors that connect only to devices with the same final objective. Thus, the connector of the enteral cable set is expected to be incompatible with the venous catheter connector. The devices also present different colors to attract the attention of the professional and avoid a possible connection error^(^ 103 ^-^ 104 ^)^.

Organizational managers and leaders should rethink the procurement processes of these devices to promote safety for tube-fed patients and reduce the costs of AE. They should also work with manufacturers to encourage the creation of new alternatives to solve the problems related to medical devices^(^ 105 ^)^.

While efforts were made to uphold rigor for an integrative review and a comprehensive literature search was done, we acknowledge that this review has some limitations. First, only those articles available for free were included. This may have resulted in the omission of several valuable studies. Second, this review did not take into consideration the characteristics of the patients with an NGT/NET such as age, inpatient unit and time of use. These factors may contribute to AEs. Last, articles with similar research that did not use our broad search criteria may have been automatically excluded during the initial search.

## Conclusion

Sixty-nine primary articles were included in this review, and AEs were mostly respiratory; in 16 articles, death was reported. Although respiratory AEs were the most common, other equally severe AEs were identified, such as 8% burned body surface due to extravasation of gastric juice, enteral feeding into the bloodstream, and organ perforations.

The results may also contribute to healthcare professionals, especially nurses, to develop evidence-based guidelines for inserting feeding tubes at patients’ bedside and for verifying feeding tubes placement in adult patients. Incorporating technological advances in patient care is not easy. These advances are fundamental tools though for the reduction of AE and for the quality and safety of the patient.

This is the first integrative review on adverse events caused by NGT/NET around the world to date. Future experimental research is needed to test the feasibility and efficiency of technologies already available to improve clinical practice and patient safety. In addition, future studies should establish the patients’ factors that may lead to NGT/NET-related AEs in order to reduce risks and improve patient outcomes.
